# Patient-reported quality-of-life outcomes after ATOMS surgery for post-prostatectomy stress urinary incontinence managed with an on-demand follow-up strategy

**DOI:** 10.1177/17562872261442654

**Published:** 2026-04-19

**Authors:** Ingunn Roth, Karin Margrethe Hjelle, Christian Beisland, Christian Arvei Moen, Patrick Juliebø-Jones

**Affiliations:** Department of Urology, Haukeland University Hospital, Bergen, Norway; Department of Clinical Medicine, University of Bergen, Bergen, Norway; Department of Urology, Haukeland University Hospital, Bergen, Norway; Department of Clinical Medicine, University of Bergen, Bergen, Norway; Department of Urology, Haukeland University Hospital, Bergen, Norway; Department of Clinical Medicine, University of Bergen, Bergen, Norway; Department of Urology, Haukeland University Hospital, Jonas Lies vei 65, 5021 Bergen, Norway

**Keywords:** adjustable sling, prostate cancer, prostheses and implants, stress urinary incontinences, surgical outcomes

## Abstract

**Background:**

Stress urinary incontinence (SUI) following radical prostatectomy (RP) can impair quality of life (QoL). While surgical treatments have shown promising short-term results, data on long-term patient-reported outcomes remain limited.

**Objectives:**

To evaluate symptom burden, perceived improvement, and health-related quality of life (HRQoL) at extended follow-up after adjustable male obturator system (ATOMS) surgery.

**Design:**

Retrospective questionnaire-based observational study.

**Methods:**

Men with post-RP SUI who underwent ATOMS implantation between 2012 and 2023 at a single tertiary centre were retrospectively identified (*n* = 111). Postoperative follow-up beyond the early postoperative period was conducted on an on-demand basis. Survivors (*n* = 99) were contacted and, following informed consent, completed the EPIC-26, RAND-12, and Patient Global Impression of Change questionnaires. Follow-up duration was defined as the time from ATOMS surgery to questionnaire completion.

**Results:**

Eighty-three patients participated (median age: 75 years; median follow-up: 87 months). At follow-up, 66% reported perceived improvement. Perceived improvement correlated with higher RAND-12 physical and mental scores. Pad use negatively correlated with both physical and mental HRQoL (*r* = −0.4, *p* < 0.001). Prior radiotherapy and higher BMI were associated with poorer EPIC-26 incontinence scores. RAND-12 scores were comparable to age-matched general population data. Device removal did not significantly impact HRQoL scores. At final follow-up, only 11% used were completely pad-free.

**Conclusion:**

ATOMS surgery was associated with symptom improvement and favourable quality-of-life outcomes for most patients. In the context of an on-demand postoperative follow-up strategy without routine scheduled refilling, continence outcomes likely reflect the management model employed, and structured reassessment and refilling may therefore serve to optimise long-term results.

## Plain language summary

Quality of life after surgery for incontinence after radical prostatectomy

This study assessed outcomes of 83 men with stress urinary incontinence (SUI) after radical prostatectomy who underwent ATOMS surgery between 2012 and 2023. 66% of patients reported perceived improvement. Improved urinary control was associated with better physical and mental quality of life, while higher BMI and prior radiotherapy predicted worse incontinence outcomes. Notably, overall health scores were comparable to the general population, and device removal did not significantly affect quality of life.

## Introduction

In the setting of men with persistent stress urinary incontinence (SUI) post radical prostatectomy (RP), the negative impact on their quality of life is recognised to be significant.^1^ Implant surgery in the form of the artificial urinary sphincter (AUS) or male slings can be indicated.^2^ In a national Swedish study (1998–2012), 3% of men underwent incontinence surgery post-RP at a median of 3 years.^3^ A similar rate of 3.4% within 5 years post-RP was reported from a US study (2003–2017) of over 20,000 men. In the latter study, male slings were the commonest (47.5%) procedure. The adjustable male obturator system (ATOMS™, A.M.I., Austria) is an example of such a compressive device.^4^ An increasing number of studies have shown that this device can yield improvement in objective outcome measures such as pad count and weight.^5^ In contrast, the impact of incontinence surgery on health-related quality of life (HRQoL), particularly from a long-term perspective, has been more lacking. This is the case for both slings and other incontinence surgeries in general, although especially among males. Our objective was to evaluate symptom burden, perceived improvement and quality of life at long-term follow-up.

## Materials and methods

As part of an ethically approved retrospective, questionnaire-based observational study (approval number REK2024/726791, May 2024), all men with SUI post-RP who underwent ATOMS surgery between January 2012 and December 2023 at a tertiary centre were identified (*n* = 111).

Inclusion criteria were men with SUI after RP who underwent ATOMS surgery within the study period. Exclusion criteria were patients who received ATOMS implantation for benign causes. No *a priori* sample size calculation was performed, as all eligible patients treated during the study period were included.

The standard indication for ATOMS is in patients with mild to moderate urinary incontinence, as defined by the 24-h pad weight test: mild (0–200 g), moderate (200–400 g), and severe (>400 g). To those who were still alive (*n* = 99), the following validated questionnaires were administered via post: Expanded Prostate Cancer Index Composite Short Form (EPIC-26) and Norwegian adapted version of the modified Patient Global Impression of Change (PGI-C) scale, based on the seven-point scale originally published by Hurst et al.^6–11^ In this version, the first two categories represent ‘no improvement’, while options 3–7 represent varying degrees of perceived improvement. This scale had already been in clinical use in Norway and was judged culturally appropriate at the time of study design. The RAND-12 was also administered, measuring health-related quality of life (HRQoL) with a physical component summary (PCS) and mental component summary (MCS). Scoring for each questionnaire/scale adhered to the specific instructions and guidance provided by the original authors. In addition, data was collected on demographics and baseline parameters at the time of incontinence surgery. Written consent was obtained for each participant. License permissions for questionnaire usage were acquired from the relevant bodies as required.^12^ If an individual did not respond to the initial invitation by post, one follow-up telephone call was made to ask if they wished to participate. The survey was not completed over the phone, even if they indicated a wish to participate. Follow-up time was defined as the interval between ATOMS surgery and questionnaire completion. The response rate was 90% (*n* = 99) with 83 patients consenting to be included in the study (Supplemental Figure 1). Outcomes for early post operative data have been published in a previous study.^13^

In accordance with local practice, all patients attended a postoperative assessment at 3 months following surgery at the tertiary centre. Beyond, this further assessment was carried out on an on-demand basis, based on clinical need and patient preferences. At the point where further follow-up was not required or requested, patients were discharged back to their local centre, with re-referral available as required. Median follow-up duration was 12 months (IQR 6–22). Forty-three (52%) patients had clinic follow-up exceeding 12 months. Overall, the median number of clinic contacts after surgery was 3 (IQR 2–4).

This study was prepared and reported in accordance with the STROBE (Strengthening the Reporting of Observational Studies in Epidemiology) guidelines^14^ (Supplemental Table 1).

### Statistical analysis

An independent *t*-test was used to compare continuous variables, or Mann-Whitney test when normality could not be assumed. Chi-squared test was applied for categorical variables, or Fisher’s exact test where appropriate. Pearson’s correlation was employed to assess the relationship between continuous variables. Spearman’s test was used instead when the data were ordinal or not normally distributed. Age-adjusted RAND scores were obtained for the Norwegian male population and compared to the study data using a one-sample *t*-test.^15^ Multivariable logistic regression was performed to identify predictors of the poorest scores (<25) for the EPIC-26 incontinence domain summary. Number of pads used was categorised into four ordinal groups according to the related EPIC-26 item (none, one pad per day, two pads per day, three or more pads per day). The findings were then compared across three time points (T1: Pre-operative, T2: Post ATOMS surgery, T3: Study follow-up). The groups were no pads, one pad, two pads and three or more pads based on the questionnaire EPIC item to this domain. The Wilcoxon signed-rank test was then employed for pairwise comparisons (T1 vs T2, T2 vs T3, T1 vs T3). Non-responder analysis was performed to compare key baseline parameters between the final study sample and the non-respondents. All analyses were done using IBM SPSS Statistics 29.0 (IBM Corp., Armonk, NY, USA) with statistical significance set by *p* < 0.05.

## Results

Across the 83 patients in the final study sample, the median age was 75 years (IQR 70–78), and the median time since ATOMS surgery was 87 months (IQR 59–111; Table 1). Twenty-eight per cent had previously undergone radiotherapy. Overall, seven patients (8%) experienced complications in the early postoperative period (<30 days), all of which were Clavien–Dindo grade I and related to pain.

**Table 1.** Baseline characteristics.

Cohort demographics	*n* = 83
Index surgery, *n* (%):	
RALP	74 (89)
ORRP	9 (11)
Prior radiotherapy, *n* (%)	23 (28)
Median age at prostate surgery, years (IQR)	65 (60–68)
Median age at ATOMS surgery, years	69 (64–73)
ASA, median (IQR)	2 (2–2)
BMI, median (IQR)	27 (25–29)
Degree of leakage (g/24 h), *n* (%):	
Mild < 100 g/24 h	29 (35)
Moderate 100–400 g/24 h	43 (52)
Severe > 400 g/24/h	10 (12)
Missing data	1 (1.2)
Number of pads/24 h, median (IQR)	3 (2–4)
Need for reoperation, *n* (%)	11 (13)

ASA, American Society of Anesthesiologists; IQR, Interquartile range; ORRP, Open radical retropubic prostatectomy; RALP, Robot assisted laparoscopic prostatectomy.

At the time of ATOMS implantation, most patients had either mild (35%) or moderate (52%) SUI, with a median pad use of 3 per 24 h. During follow-up, 11 patients (13%) underwent reoperation: five due to recurrent SUI, for which they received an artificial urinary sphincter, and six due to port-related complications (five relocations and one removal).

Summary scores for the questionnaires administered are detailed in Table 2. According to PGI-C, 66% recorded perceived improvement of varying levels as result of their surgery.

**Table 2.** Summary of questionnaire scores.

EPIC-26	
HRQoL domain summary scores	Median (IQR)
Urinary: incontinence	38 (15–53)
Urinary: irritative/Obstructive	82 (69–94)
Bowel	96 (79–100)
Sexual	13 (6–21)
Hormonal	85 (75–96)
RAND-12	
Domain summary scores	Mean (range, SD)
PCS12	66 (6–100, 25)
MCS12	73 (13–100, 24)
PGI_C	
Response	*N* (%)
No change or worse	14 (16.9)
Like before	14 (16.9)
Some improvement	4 (4.8)
A little better	7 (8.4)
Moderately better	8 (9.6)
Much better	27 (32.5)
Very much better	8 (9.6)
Missing	1 (1.2)

HRQoL, health-related quality of life.

Mental health scores as indicated by RAND-12 MCS were higher in those patients with perceived improvement (75 vs 68, *p* = 0.3) as was physical health according to RAND-12 PCS (71 vs 58, *p* = 0.04). A positive correlation was found between the RAND-12 MCS and PCS and the EPIC-26 incontinence domain summary score (*r* = 0.40, *p* = 0.001). There was no correlation found between time since ATOMS surgery and incontinence domain scores (*r* = 0.1, *p* = 0.5). Based on the individual EPIC item for pad use, there was a negative correlation between reported number of pads used and MCS (*r* = −0.4, *p* < 0.001) as well as PCS (*r* = −0.4, *p* < 0.001). Simple linear regression revealed pad use to significantly predict MCS (*B* = −10, β = −0.4, *p* < 0.001). This indicated that for each additional pad used, MCS decreased by 10 points. The same was found for PCS (*B* = −10, β = −0.4, *p* < 0.001). While MCS were lower if implant had been removed, the mean difference was not significant (36 vs 39, *p* = 0.7). The findings were similar for PCS (31 vs 41, *p* = 0.2). EPIC-26 incontinence domain summary scores were significantly lower if the patient had previously undergone radiotherapy (28 vs 44, *p* = 0.006). This was the same for the EPIC irritative/obstructive domain (28 vs 43, *p* = 0.01) and EPIC bowel domain summary scores (27 vs 44, *p* = 0.002). The difference was lower but not significant for EPIC hormone domain summary (32 vs 42, *p* = 0.9) and EPIC sexual domain summary (39 vs 40, *p* = 0.7). Responses for the EPIC items that make up the incontinence domain are summarised in Table 3.

**Table 3.** Distribution of responses for EPIC-26 urinary incontinence domain items.

EPIC-26 item	Response option 1	Response option 2	Response option 3	Response option 4	Response option 5
Item 23. Frequency of urine leakage (past 4 weeks)	More than once a day (67.9%)	About once a day (11.1%)	More than once a week (8.6%)	About once a week (7.4%)	Rarely or never (4.9%)
Item 26. Urinary control (past 4 weeks)	No control whatsoever (19.8%)	Frequent dribbling (28.4%)	Occasional dribbling (48.1%)	Total control (3.7%)	
Item 27. Pads per day (past 4 weeks)	None (10.8%)	1 pad (31.3%)	2 pads (26.5%)	3+ pads (31.3%)	
Item 28a. Problem: dripping/leaking urine (past 4 weeks)	No problem (2.5%)	Very small problem (14.8%)	Small problem (23.5%)	Moderate problem (37%)	Big problem (22.2%)

At follow-up, 11% used zero pads and 31% were using one pad daily (Table 4). When comparing time points, there was a significant reduction in pad count between T1 and T2 (*Z* = −7.12, *p* < 0.001). While there was an increase in pad use between T2 and T3 (*Z* = +5.5, *p* < 0.002), a reduction persisted when comparing T1 and T3 (*Z* = −5.4, *p* < 0.002), with a median pad use of two pads at T3.

**Table 4.** Comparison of pad count across study points.

Time point	T1: pre-op	T2: 3 months post operative	T3: study follow-up
0 pads (%)	0	52.5	10.8
1 pad (%)	9.6	23.8	31.3
2 pads (%)	22.9	12.5	26.5
3+ pads (%)	67.5	11.3	31.3

Multivariable logistic regression identified only BMI as a significant predictor of the lowest EPIC incontinence domain summary scores (*p* = 0.02; Table 5). Compared to reference data for an age-adjusted Norwegian male population, men who underwent ATOMS surgery showed lower but not significantly different PCS (66 vs 70, *Z* = −0.13, 95% CI −8.9, 2.4, *p* = 0.1) or MCS (73 vs 77, *Z* = −0.18, 95% CI −10, 1.0, *p* = 0.1) scores.

**Table 5.** Multivariable logistic regression for predictors of poor EPIC incontinence domain summary scores (<25).

Characteristic	Multivariable		
	OR	95% CI	*p*-Value
Age at ATOMS	1.0	0.92, 1.08	0.9
Radiotherapy	1.75	0.55, 5,6	0.3
Time between surgeries	0.95	0.82, 1.1	0.5
Pad weight	1.00	1.00, 1.01	0.08
Body mass index	1.26	1.03, 1.53	0.02*

CI, confidence interval; OR, odds ratio.

* statistically significant value.

Non-responder analysis revealed no difference in median pad count (3 vs 3, *p* = 0.7), however, reoperation rate was lower in the study sample (12.2% vs 36.8%, *p* = 0.02).

## Discussion

In this study, two-thirds of patients reported perceived improvement following ATOMS implantation. This group also had higher RAND-12 physical and mental component scores, which indicates that this surgical intervention can have a clinically meaningful impact on a patient’s daily functioning and psychological well-being. Although the difference in mental health scores did not reach statistical significance between those who perceived improvement and those who did not, the lower scores appear consistent with existing evidence that SUI can have profound psychosocial consequences, including anxiety, depression, and social withdrawal.^16^

Pad use was significantly associated with HRQoL. Our findings reveal a strong negative correlation between the number of pads used and both RAND-12 mental and physical health scores. This supports the premise that the burden of pad use is closely bound with a patient’s lived experiences. The use of pads can be linked to a range of negative emotions, such as shame and embarrassment, as well as practical challenges in daily life, including cost and physical issues like skin irritation. Incontinence surgery has the ability to alleviate, and potentially eliminate, many of these sequelae. From an environmental aspect also, it also helps to alleviate the waste disposal burden associated with disposable products, which are largely plastic based and go to landfill.^17^ The change in RAND-12 scores of 10 points can be considered to be a clinically important difference (CID) as has been reported in previous studies.^18^

Although the RAND-12 scores of our cohort were slightly lower than those of the age-matched Norwegian male population, the differences were not statistically significant. This suggests that, for many patients, ATOMS surgery enables a return to a level of physical and mental health comparable to the general population, a reassuring finding given the substantial impairment on QoL often reported among those with SUI post-RP.^19^

Despite initial improvements in continence following ATOMS implantation, an increase in pad use was observed between the early postoperative phase (T2) and long-term follow-up (T3). The pad-free rates observed in our cohort were lower than those reported in a meta-analysis, where Esquinas et al.^20^ described a pooled dryness rate of 67% and a 90% improvement rate after adjustment across 1,393 patients. Similarly, in a cohort of 99 patients, Giammò et al.^20,21^ reported 45% social continence and 74.7% self-reported continence at a comparable follow-up. It is important to note, however, that many of these series originate from centres employing structured postoperative reassessment and scheduled refilling protocols. In contrast, adjustments in our setting were performed on an on-demand basis without routine long-term reassessment. As such, the continence outcomes observed here likely reflect the management model employed rather than intrinsic device performance alone. Structured postoperative reassessment and refilling may therefore play an important role in optimising long-term continence.

Prior radiotherapy was associated with poorer outcomes across multiple EPIC-26 domains, including incontinence, irritative/obstructive symptoms, and bowel function. These findings are consistent with previous reports that patients with a history of pelvic radiotherapy tend to experience more complex incontinence patterns and may respond less favourably to sling procedures.^22^ Similarly, higher BMI emerged as a predictor of the lowest EPIC incontinence scores. This highlights the potential influence of obesity on pelvic floor support and continence, and thus reinforces the value of pre-operative counselling and consideration of pre-operative weight management interventions. It also emphasises how patient-related factors can also have bearing on long-term functional success. Device-related complications were relatively infrequent and while those with device removal had lower quality of life scores, the differences were not statistically significant. This may reflect either resilience or adaptation among these patients, or alternatively, the limited sample size may have reduced the statistical power.

## Strengths and limitations

There are a number of limitations in this study to acknowledge. Given postoperative management based on an on-demand strategy rather than regular, periodically scheduled long-term follow-up, the findings should be interpreted within this clinical context. The questionnaires in this study captured responses from a single time point only. Administration at the time of surgery as well would have allowed for changes over time to be measured. In addition, objective outcomes such as pad weight at follow-up have not been included. The results also represent findings from a single centre only, which can limit external validity. Response bias should also be acknowledged, in that the reoperation rate was lower in the final study sample, which could lead to outcomes being more favourable and less generalisability. The absence of a power analysis may limit the statistical strength of subgroup comparisons. EPIC-26 does not differentiate between stress, urgency, or mixed urinary incontinence, which limits the interpretation of postoperative outcomes. As most patients were overweight, the applicability of the findings to men with body mass index values within the normal reference range may be limited.

Although some patients underwent surgery toward the end of the study period and consequently had shorter follow-up, the overall median follow-up for the cohort exceeded 7 years. An additional strength of the study was the high response rate, which surpassed 90%. We present data in a group of patients where l follow-up data in terms of subjective outcomes, particularly following ATOMS surgery, is scarce. We included only patients with SUI post-RP, as opposed to all causes, to reduce heterogeneity. Patients did not receive payment, and questionnaires were not filled out by a health professional on the phone, which is a method that can introduce bias. Future studies should aim to implement these questionnaires in a prospective format as part of a multicentre study setting, which would allow for serial measurements over time to be assessed. Development of a dedicated patient-reported outcome measure tool would also complement this.

## Conclusion

ATOMS surgery was associated with symptom improvement and favourable health-related quality-of-life outcomes for most patients. Importantly, continence outcomes in this cohort were observed within the context of an on-demand postoperative follow-up strategy without routine scheduled refilling. As such, these results likely reflect the management model employed rather than intrinsic device performance alone. Structured postoperative reassessment and refilling may well therefore serve to optimise long-term continence outcomes.

## Supplemental Material

sj-docx-1-tau-10.1177_17562872261442654 – Supplemental material for Patient-reported quality-of-life outcomes after ATOMS surgery for post-prostatectomy stress urinary incontinence managed with an on-demand follow-up strategy Supplemental material, sj-docx-1-tau-10.1177_17562872261442654 for Patient-reported quality-of-life outcomes after ATOMS surgery for post-prostatectomy stress urinary incontinence managed with an on-demand follow-up strategy by Ingunn Roth, Karin Margrethe Hjelle, Christian Beisland, Christian Arvei Moen and Patrick Juliebø-Jones in Therapeutic Advances in Urology

sj-docx-2-tau-10.1177_17562872261442654 – Supplemental material for Patient-reported quality-of-life outcomes after ATOMS surgery for post-prostatectomy stress urinary incontinence managed with an on-demand follow-up strategy Supplemental material, sj-docx-2-tau-10.1177_17562872261442654 for Patient-reported quality-of-life outcomes after ATOMS surgery for post-prostatectomy stress urinary incontinence managed with an on-demand follow-up strategy by Ingunn Roth, Karin Margrethe Hjelle, Christian Beisland, Christian Arvei Moen and Patrick Juliebø-Jones in Therapeutic Advances in Urology

## Declarations

**Ethics approval and consent to participate:** Formal approval received from the Western Norway Regional Health Authority Ethical Committee. Approval number REK2024/726791, May 2024). All participants provided written informed consent.

**Consent for publication:** As part of the written consent, patients also provided consent for publication.

**Availability of data and materials:** Data is available upon reasonable request to the corresponding author*. Of note, copyright and licensing restrict making the validated questionnaires available as a Supplemental File. Further details of the questionnaire can be received by contacting the corresponding author.
