# Supplementary figures and images for: Patient-reported quality-of-life outcomes after ATOMS surgery for post-prostatectomy stress urinary incontinence managed with an on-demand follow-up strategy
Source: Ther Adv Urol. 2026 Apr 19;18:17562872261442654. doi: 10.1177/17562872261442654 (PMC13110293; doi:10.1177/17562872261442654)

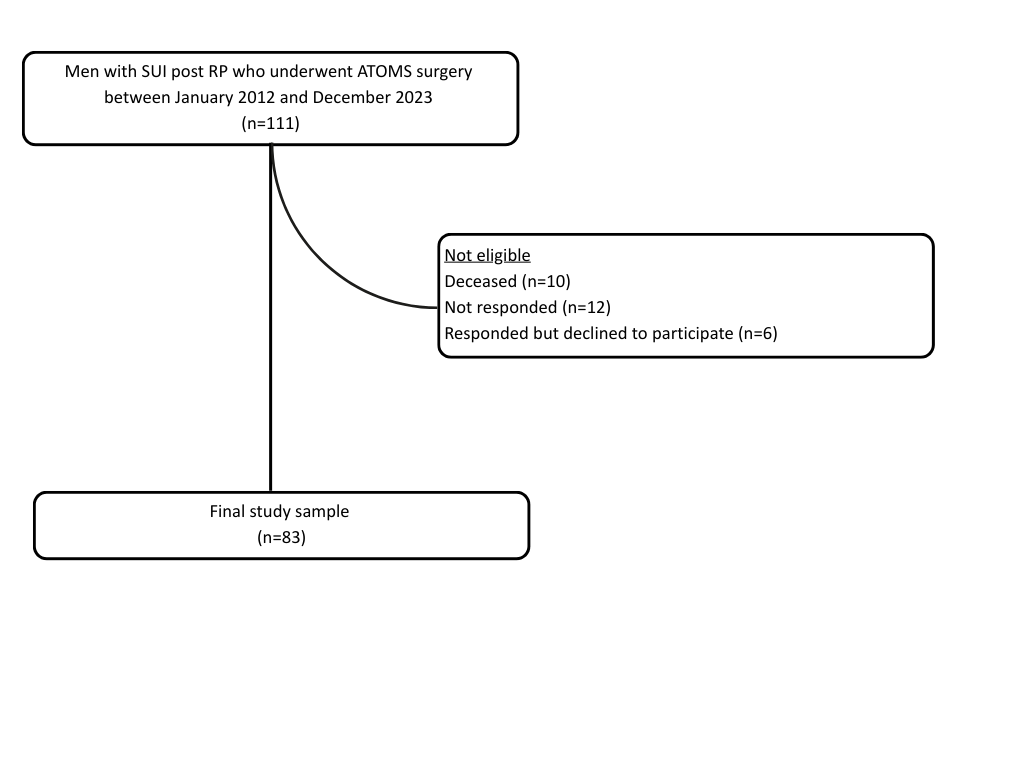


Supplementary figure 1. Flow diagram for study sample inclusion

Supplement: sj-docx-1-tau-10.1177_17562872261442654 – Supplemental material for Patient-reported quality-of-life outcomes after ATOMS surgery for post-prostatectomy stress urinary incontinence managed with an on-demand follow-up strategy [file sj-docx-1-tau-10.1177_17562872261442654.docx]
