# Supplementary material for: Patient-reported quality-of-life outcomes after ATOMS surgery for post-prostatectomy stress urinary incontinence managed with an on-demand follow-up strategy
Source: Ther Adv Urol. 2026 Apr 19;18:17562872261442654. doi: 10.1177/17562872261442654 (PMC13110293; doi:10.1177/17562872261442654)
Supplement: sj-docx-2-tau-10.1177_17562872261442654 – Supplemental material for Patient-reported quality-of-life outcomes after ATOMS surgery for post-prostatectomy stress urinary incontinence managed with an on-demand follow-up strategy [file sj-docx-2-tau-10.1177_17562872261442654.docx]

# Supplementary File 1. STROBE Checklist

| Item No. | STROBE Recommendation | Section of Manuscript Where Addressed |
| --- | --- | --- |
| 1a | Indicate the study design in the title or abstract. | Title and Abstract |
| 1b | Provide an informative and balanced summary of what was done and what was found. | Abstract |
| 2 | Explain the scientific background and rationale for the investigation. | Introduction |
| 3 | State specific objectives, including any prespecified hypotheses. | Introduction, final paragraph |
| 4 | Present key elements of study design early in the paper. | Materials and Methods, first paragraph |
| 5 | Describe the setting, locations, and relevant dates, including periods of recruitment and follow-up. | Materials and Methods |
| 6a | Give the eligibility criteria, and the sources and methods of selection of participants. | Materials and Methods (Inclusion and exclusion criteria) |
| 6b | For matched studies, give matching criteria and numbers of exposed and unexposed. | Not applicable |
| 7 | Clearly define all variables, outcomes, exposures, predictors, potential confounders, and effect modifiers. | Materials and Methods |
| 8 | For each variable, give sources of data and details of methods of assessment (measurement). | Materials and Methods |
| 9 | Describe any efforts to address potential sources of bias. | Strengths and Limitations section |
| 10 | Explain how the study size was arrived at. | Materials and Methods (All eligible patients included) |
| 11 | Explain how quantitative variables were handled in the analyses. If applicable, describe which groupings were chosen and why. | Statistical Analysis section |
| 12a | Describe all statistical methods, including those used to control for confounding. | Statistical Analysis |
| 12b | Describe any methods used to examine subgroups and interactions. | Statistical Analysis (BMI, radiotherapy subgroups) |
| 12c | Explain how missing data were addressed. | Discussion (response bias noted) |
| 12d | If applicable, describe analytical methods taking account of sampling strategy. | Not applicable |
| 12e | Describe any sensitivity analyses. | Not performed |
| 13a | Report numbers of individuals at each stage of study. | Results (n=111 eligible, n=99 contacted, n=83 included) |
| 13b | Give reasons for non-participation at each stage. | Results (non-responder analysis) |
| 13c | Consider use of a flow diagram. | Supplementary Figure 1 |
| 14a | Give characteristics of study participants and information on exposures and potential confounders. | Results, Table 1 |
| 14b | Indicate number of participants with missing data for each variable of interest. | Complete data for included participants |
| 15 | Report numbers of outcome events or summary measures. | Results (Tables 2–4) |
| 16a | Give unadjusted and adjusted estimates and their precision (e.g., 95% CI). | Results (logistic regression findings) |
| 16b | Report category boundaries when continuous variables were categorized. | Materials and Methods (pad use categories) |
| 16c | If relevant, consider translating estimates of relative risk into absolute risk. | Not applicable |
| 17 | Report other analyses done (subgroups, interactions, sensitivity analyses). | Results (radiotherapy, BMI) |
| 18 | Summarize key results with reference to study objectives. | Discussion, first paragraph |
| 19 | Discuss limitations of the study. | Strengths and Limitations |
| 20 | Give a cautious overall interpretation of results. | Discussion and Conclusion |
| 21 | Discuss the generalizability (external validity) of the study results. | Strengths and Limitations |
| 22 | Give the source of funding and the role of the funders. | Not applicable (no external funding) |
